# Realizing the gravity of the simulation: adaptation to simulated hypogravity leads to altered predictive control

**DOI:** 10.3389/fphys.2024.1397016

**Published:** 2024-05-24

**Authors:** Chase G. Rock, Samuel T. Kwak, Angela Luo, Xiao Yang, Kristy Yun, Young-Hui Chang

**Affiliations:** Comparative Neuromechanics Laboratory, School of Biological Sciences, Georgia Institute of Technology, Atlanta, GA, United States

**Keywords:** motor learning, motor control, gravity adaptation, muscle preactivation, locomotion, biomechanics

## Abstract

Accurate predictive abilities are important for a wide variety of animal behaviors. Inherent to many of these predictions is an understanding of the physics that underlie the behavior. Humans are specifically attuned to the physics on Earth but can learn to move in other environments (e.g., the surface of the Moon). However, the adjustments made to their physics-based predictions in the face of altered gravity are not fully understood. The current study aimed to characterize the locomotor adaptation to a novel paradigm for simulated reduced gravity. We hypothesized that exposure to simulated hypogravity would result in updated predictions of gravity-based movement. Twenty participants took part in a protocol that had them perform vertically targeted countermovement jumps before (PRE), during, and after (POST) a physical simulation of hypogravity. Jumping in simulated hypogravity had different neuromechanics from the PRE condition, with reduced ground impulses (*p* ≤ .009) and muscle activity prior to the time of landing (i.e., preactivation; *p* ≤ .016). In the 1 g POST condition, muscle preactivation remained reduced (*p* ≤ .033) and was delayed (*p* ≤ .008) by up to 33% for most muscles of the triceps surae, reflecting an expectation of hypogravity. The aftereffects in muscle preactivation, along with little-to-no change in muscle dynamics during ground contact, point to a neuromechanical adaptation that affects predictive, feed-forward systems over feedback systems. As such, we conclude that the neural representation, or internal model, of gravity is updated after exposure to simulated hypogravity.

## Introduction

An animal’s predictions regarding movement through their environment are an essential aspect of motor control. From a predator’s ability to anticipate their prey’s trajectory (Ben-Tov et al., 2018) to a person’s ability to catch a ball (Savelsbergh et al., 1992), predictions facilitate successful movement. Such predictions are especially interesting from a motor control perspective, as they reveal the assumptions that are being employed by the nervous system. For example, a bat’s ability to accurately approach its prey via echolocation demonstrates a consistent, innate understanding of the speed of sound (Amichai and Yovel, 2021). Similarly, people employ predictions of physics to perform tasks as simple as intercepting a ball (Bosco et al., 2012; La Scaleia et al., 2015) or as complicated as assessing whether or not a given stack of objects will fall over (Battaglia et al., 2013; Ullman et al., 2017). These predictive abilities have been attributed to a neural model of the physics involved in each situation (Battaglia et al., 2013; Ullman et al., 2017), with some specific attention on the effects of gravity (Zago et al., 2009). Such neural models are referred to as “internal models,” and so for this paper we will refer to the *internal model of gravity* as the neural processes that underpin predictions of gravity-based motion.

The human neuromuscular system employs a robust internal model of gravity (Jörges and López-Moliner, 2017; Rousseau et al., 2021), especially for tasks that involve projectile motion (Zago et al., 2009). For example, when intercepting a ball, muscles of the arm are activated prior to the ball making contact, demonstrating a preparatory activation that relies on a prediction of gravity (Zago et al., 2005). Furthermore, when a similar catching task is performed in microgravity, the arm muscles are still activated as if normal Earth’s gravity were still present (i.e., the movement begins too early), even after weeks of experience in microgravity (McIntyre et al., 2001). The maintenance of an expectation of Earth’s gravity in the absence of gravity-related sensory cues indicates a feed-forward internal model of gravity is being used by the neuromuscular system as opposed to a purely feedback model in which error-based corrections are made. This evidence from exposure to spaceflight (McIntyre et al., 2001), along with ground-based investigations of simulated microgravity (Zago et al., 2005), indicates an internal model of gravity that reliably expects Earth’s gravity.

However, there is also evidence indicating an adaptability of the internal model of gravity. Firstly, humans are not born with a robust understanding of gravity; in fact, it takes years of development before a person accurately expects the effect of gravity on a projectile (Kim and Spelke, 1999). Because the internal model of gravity is learned over time (i.e., not innate), it is likely open to adaptation. Secondly, locomotion on the lunar surface (Minetti et al., 2012), as well as in simulated hypogravity environments on Earth (Cavagna et al., 1998; Lacquaniti et al., 2017), has revealed altered strategies for successfully moving within new gravity levels. Similarly, interactions with projectiles on a virtually simulated Mars demonstrate that people can adapt to and predict the effects of Martian gravity (Torok et al., 2019). While hypogravity (0 < g < 9.81 m/s^2^) represents a different challenge from microgravity (g ≈ 0.0 m/s^2^), adaptability in the face of new gravity levels appears to contrast with the idea of a robust, seemingly constant internal model of Earth’s gravity (McIntyre et al., 2001). To reconcile this simultaneous flexibility and rigidity of the internal model of gravity, we looked to test the adaptability of a locomotor task that clearly relies on the predictions provided by an internal model of gravity.

We aimed to characterize adaptation of the internal model of gravity in response to countermovement jumps in simulated hypogravity. Operating under the hypothesis that the internal model of gravity would adapt to the new gravity level, we predicted that behavior during and immediately after simulated hypogravity exposure would reflect the feedforward expectation of hypogravity. In the current study, participants performed vertically targeted jumps in normal gravity and in a simulated hypogravity setting. By investigating movement at multiple time points during hypogravity adaptation and following a return to normal gravity, we shed light on an adaptability to new gravity levels that is consistent with an updated internal model of gravity.

## Materials and methods

### Participants

Twenty-five participants provided informed consent according to a protocol approved by the Georgia Institute of Technology Institutional Review Board. Two participants were excluded from all analyses for regularly exceeding the vertical limits of the reduced gravity simulator, thereby experiencing short intervals of normal gravity in the hypogravity condition. EMG data were missing for three additional subjects, leaving 20 healthy participants whose data are presented in the present study (12 female, 8 male; Height: 170.2 ± 8.8 cm; Mass: 61.2 ± 11.6 kg; Age: 23.2 ± 6.0 years). Sixteen participants self-reported that they were right-handed and four reported left-hand dominance. By observing the foot each participant used to kick a ball (van Melick et al., 2017), we determined that eighteen participants were right-foot dominant and two were left-foot dominant.

### Equipment

Kinematic and kinetic data were collected via a 12-camera 3D motion analysis system (Vicon Motion Systems, Oxford, UK) and two floor-embedded force plates (AMTI, Watertown, MA). An EMG electrode system (Motion Lab Systems, Baton Rouge, LA) was used to obtain muscle activity measurement from the dominant and non-dominant side triceps surae group: specifically, the Soleus muscle (SOL), Medial Gastrocnemius muscle (MG), and Lateral Gastrocnemius muscle (LG). Data were collected and synchronized through Vicon Nexus, processed in Visual3D (C-Motion, Germantown, MD), and final calculations and comparisons were performed using custom MATLAB scripts. Ten of the twenty participants were also fitted with an ultrasound probe (TELEMED, Vilnius, LTU) to measure the muscle fascicle dynamics of the MG muscle.

**FIGURE 1:**
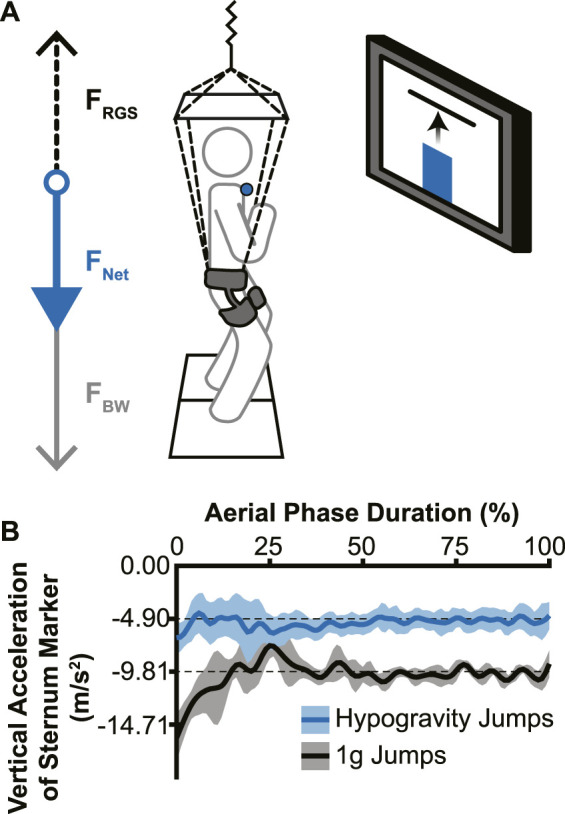
Experimental setup and effectiveness of the reduced gravity simulator (RGS). **(A)** The RGS was attached to a hip-worn body harness and pulled up (dotted black arrow; F_RGS_) with a constant force ranging from 37% to 59% of the participant’s body weight (grey arrow; F_BW_), resulting in a reduced net downward force (blue arrow; F_NET_). Live visual feedback was shown to the participant via a monitor directly in front of them, with the vertical position of the sternum marker (blue circle) represented by a vertical bar (blue bar) and the target height represented by a horizontal line. **(B)** Example vertical accelerations during the aerial phase from a single participant’s sternum marker. Jumps without the RGS (black line) confirmed a normal acceleration due to Earth’s gravity and hypogravity jumps (with RGS; blue line) showed a reduced acceleration that accurately simulated −0.5 g for this participant (lines indicate mean acceleration across jumps in each condition; shading indicates ± 1 standard deviation).

The custom reduced gravity simulator (RGS) consisted of a set of constant-force springs (MacLean and Ferris, 2020) that pulled upward on a modified rock-climbing harness worn by the participant near their body center of mass. The constant-force springs were attached to the harness to apply a force near the body center of mass that supported a portion of the participant’s body weight, effectively simulating a hypogravity environment. An overhead aluminum frame was used to guide the load-bearing lines away from the head, arms, and torso (Chang et al., 2000). Vertical acceleration of retroreflective markers on the trunk were assessed to confirm that the RGS imposed a consistent change in acceleration (Figure 1B).

The visual feedback system used for the experiment consisted of a 50” monitor directly in front of the participant at approximately eye-level with the target height displayed as a red horizontal line against a white background. The participants’ real-time height relative to the target was represented as a vertical bar on the screen that rose and fell with their movements (Figure 1A).

### Experimental set-up

After completion of consent and questionnaire, participants donned the modified rock-climbing harness, which experimenters adjusted for participant comfort. The skin overlying each muscle belly was prepped for EMG electrode placement by shaving any hair and rubbing briskly with an alcohol wipe to reduce impedance. EMG electrodes were placed and tested for signal quality. A retroreflective marker for the visual feedback system was placed on the sternal notch, with additional markers placed on vertebra C7, clavicle (left and right side), and scapula (left and right side) to facilitate motion tracking of the sternal notch.

For the 10 participants on whom ultrasound data were collected, the ultrasound probe was placed with a custom gel mold and secured in place with an elastic wrap. We placed the probe on the distal end of the MG on the dominant leg so that the muscle-tendon junction (MTJ) was visible throughout the full range of ankle flexion. When turned on, the ultrasound scanner sent a trigger signal to the motion analysis system which was used to time sync the ultrasound images to all other data after adjusting for the delay between the trigger output and the initial ultrasound frame. For these participants, additional markers were placed on the ultrasound probe as well as the knee, shank, insertion point of the Achilles’ tendon, and foot.

Each experimental session started with a calibration trial that measured standing reference height as determined by the sternal notch marker and body weight. Participants then performed three maximum jumps with their arms crossed to minimize any arm movements relative to the body center of mass. Participants were allowed at least 30 s of rest between maximum jumps to minimize fatigue. The target height was set at 75% of the maximum vertical displacement during the participant’s highest maximum jump.

### Experimental protocol

During a series of pre-adaptation jumps, the participants jumped ten times at unaltered gravity (i.e., 1 g), with their arms held across their chest towards the goal of reaching the virtual target as accurately as possible. The subjects jumped once for every time a lab member said “Go” and were allowed to rest for as long as needed between jumps. Participants were asked to try to refrain from taking any extra steps before or after the jump. Though not specifically instructed, all participants used a countermovement leading into each jump.

After completion of the ten pre-adaptation jumps, the participants were attached to the RGS with the appropriate pulling force and their resultant vertical ground reaction force was measured to confirm the pulling force. Participants jumped to the same target 50 times in simulated hypogravity with their arms crossed with the same instructions as the pre-adaptation jumps. Following the 50 hypogravity adaptation jumps, participants were detached from the RGS and were instructed to immediately perform ten post-adaptation jumps at 1 g. Minimal movement was allowed between detaching the RGS and commencing with the POST jump to prevent adaptation washout before attempting post-training jumps.

### Data analysis: mechanics

The ground reaction forces contributing to the lift and landing of the countermovement jump were used to calculate the lift and landing impulses. The lift phase began when the vertical ground reaction force (GRFz) equaled body weight prior to reaching its peak during push-off (Mizuguchi et al., 2015) and ended with lift-off from the ground. Similarly, the land phase began with touchdown and ended at the point where GRFz crossed body weight after reaching its peak just after landing. During these time windows, the area under the GRFz curve was calculated, resulting in the lift and landing impulses.

### Data analysis: electromyography

We applied lowpass filters to the retroreflective marker (25 Hz) and ground reaction force signals (25 Hz). EMG signals were lowpass (450 Hz) and highpass (5 Hz) filtered and rectified. We then used a 100 ms sliding window average of each EMG signal on the maximum jump trials to determine the peak activation for each muscle. The peak activation for each muscle was used to normalize EMG data in all subsequent experimental trials for each participant.

To determine the beginning and end of the aerial phase of the jump, we employed a threshold of 25 Newtons of total vertical ground reaction force. We determined muscle preactivation onset time for each muscle using a method developed by Santello and McDonagh (1998), which employs a cumulative integration of the rectified EMG to determine the time at which electrical activity most rapidly increases (i.e., preactivation onset) relative to the time of landing. We made three modifications to this calculation to accommodate the low muscle activation levels during and after exposure to simulated reduced gravity. First, we investigated the overall shape of the aerial EMG trace for changes in activity by performing a lowpass filter with a cutoff frequency of 1 Hz, and local maxima were extracted from the resulting signal. If the latest occurring local maximum in the smoothed EMG signal did not occur within 150 ms of landing, we did not include it in our analysis. Second, if the timing of preactivation was calculated to be more than 150 ms prior to landing, we did not include it in our analysis, as this is outside the range of previously reported preactivation times and eliminated values that occurred far too soon (e.g., during aerial ascent). Lastly, if the average magnitude of activity over the determined preactivation phase was less than 1% of the maximum EMG activity for that muscle, we did not include it in our analysis as this represents an absence of muscle preactivation. Average EMG (i.e., preactivation magnitude) was calculated as the mean rectified EMG signal between preactivation onset time and ground contact for each jump. Over the same time period, the rectified EMG signal was integrated for each muscle and this integrated value was averaged across the three muscles of the triceps surae (MG, LG, and SOL), resulting in a comprehensive metric that reports the preactivation of the triceps surae as a whole (i.e., Triceps Surae Preactivity).

### Data analysis: muscle fascicle dynamics

The ultrasound images were exported to an image analysis software (FIJI) where the MTJ was manually tracked to generate its 2-D position (Schindelin et al., 2012). These data were interpolated and transposed into the 3-D data space and time based on the MTJ position relative to retroflective markers placed on the ultrasound probe. Because ultrasound images are produced using a rolling shutter, meaning each column of pixels represent sequential points in time, we used the horizontal position of the MTJ relative to the image width to further adjust the time delay between the ultrasound and kinematic data on a per frame basis prior to interpolation. We defined the MTJ position as the orthogonal projection of the transposed MTJ position onto the MTU. The MG muscle length was therefore the distance between the knee joint center and MTJ, and the Achilles tendon length was the distance between the MTJ and the tendon insertion. We filtered the muscle, tendon, and MTU length data using a lowpass Butterworth filter with a 6 Hz cutoff frequency. We calculated the MG muscle velocity as the first derivative of the muscle length with respect to time.

### Data analysis: muscle proprioception analysis

Muscle proprioceptive feedback from Ia afferents was estimated based on the following equation used in previous studies (Prochazka, 1999; Prilutsky et al., 2016):

$$R_{Ia}=65{\left(\frac{V_{MG}}{L_{0}}\right)}^{0.5}+200\left(L_{MG}-L_{0}\right)+k_{u}^{\max}u+R_{{Ia}_{0}}$$

R_Ia_ is the firing rate for group Ia afferents. V_MG_ is the lengthening velocity of the MG muscle, L_MG_ is the length of the MG muscle, L_0_ is the length of the MG muscle at rest. R_Ia0_ is the mean afferent firing rates at rest, but without the ability to obtain participant-specific firing rates, this term was set to zero for all calculations. The percentage maximal recruitment possible for a muscle (100 in this case) is represented by k_*u*_^*max*^ and u is the EMG muscle activity relative to maximal activation as determined by our maximum jumps which, combined with k_*u*_^*max*^, represents the occurrence of alpha-gamma coactivation which acts to stretch muscle spindles and modulate the feedback response to stretch. Muscle proprioception from force feedback was represented by the MG muscle force which was estimated as the force required to produce the calculated ankle torque, based on inverse dynamics, using the moment arm created by the orthogonal line between ankle joint center and the MTU.

### Statistical analysis

For kinematic, kinetic, and muscle activity variables, a two-tailed Student’s paired *t*-test (alpha = .05) was used to compare the PRE jump to the first hypogravity jump (Early Adaptation), the last hypogravity jump (Late Adaptation), the POST jump (POST), and the final jump (Washout). Assumptions of normality and equal variances were tested using Shapiro-Wilk’s test (Öner and Deveci Kocakoç, 2017) and Levene’s test, respectively. If either assumption was shown to be violated, a Wilcoxon Signed-Rank test was performed in place of the Student’s t-test. Effect size was estimated using Hedge’s *g*_*av*_ (Lakens, 2013).

Ultrasound data were analyzed only for PRE and POST trials. To compare the difference between PRE and POST, we used a wavelet-based functional paired *t*-test in MATLAB software, which was adapted from the wavelet-based functional ANOVA (McKay et al., 2013). This involved first performing a wavelet decomposition which required time-normalizing the data to either a factor of 2 closest to the number of frames captured for each phase or, if this decimation did not yield a wavelet coefficient that can sufficiently characterize the waveform of the data, the least number of time points needed to produce a wavelet coefficient. In this latter case, within the wavelet decomposition MATLAB function, the signal was resampled to a factor-of-2 length, decomposed, then restored back to its previous length. As a result, the lift and land phases were normalized to 64 time points and the aerial phase was normalized to 34 time points. After time-normalizing, the signals were transformed to the wavelet domain, decomposed, and analyzed using a paired, two-tailed *t*-test, comparing PRE and POST (alpha = .05). If a coefficient was determined to be significant, a contrast value was given, which is the mean difference between the two compared groups. This data was then transformed back into the time domain. Significance in the time domain was then indicated when the contrast was ≥10% of the peak contrast value and verified manually.

## Results

### Effect of simulated hypogravity on jump height, target error, and kinetics

**FIGURE 2:**
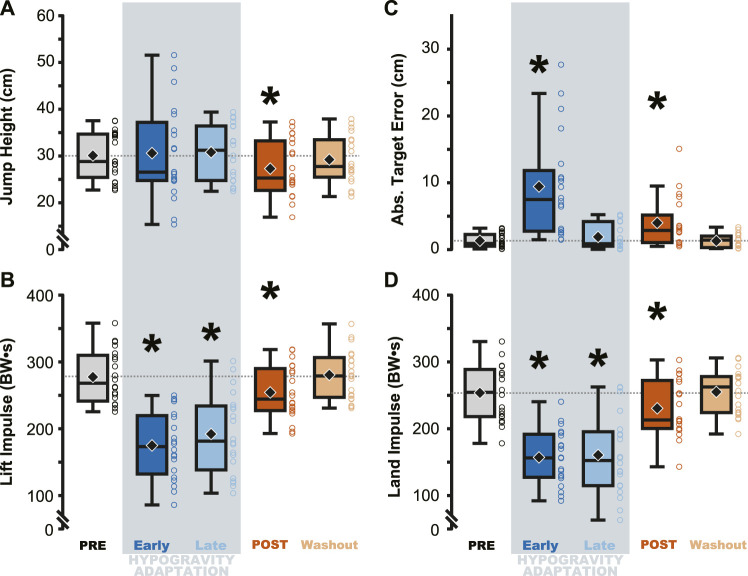
Performance on the targeted vertical jumping task was affected during and immediately following hypogravity exposure. Jumping height and average target error was more variable during early exposure to simulated reduced gravity (dark blue) but steadied by the final LOWG jump (light blue). After returning to normal gravity (POST; dark orange), participants jumped significantly lower than prior to hypogravity exposure (PRE; grey) **(A)**, with increased target error **(C)**, and reduced lift and landing impulses **(B, D)**. Performance on all four metrics were not different than PRE by the final jump in normal gravity (Washout; light orange). Boxplots represent the median (black line), mean (black diamond), interquartile ranges (top and bottom of each box), and range (whiskers) for each jump depicted across all participants. Open circles represent individual participant data for each trial. Horizontal dashed line represents the PRE mean for each metric. * indicates significant difference from PRE.

Jump performance was significantly impacted by temporary exposure to simulated hypogravity (M ± SD; 0.51 ± 0.06 g), especially upon initial exposure (Early Adaptation, Table 1 and Figure 2) and after exposure, upon returning to normal gravity (POST, Table 1 and Figure 2; Figure 7). Prior to simulated hypogravity exposure, participants jumped to the target with an average error of 1.3 ± 1.0 cm. Initial exposure to simulated hypogravity resulted in increased target error but by the final jump in simulated hypogravity target error was not different from the baseline condition (Early & Late Adaptation, Table 1 and Figure 2C). The POST jump showed a reduction in jump height, reflected by an increase in target error relative to PRE (Table 1; Figure 2A, 7A). The kinetic features of the jump match the results from jump height, with reductions in the lift and landing impulses of the jumps in simulated hypogravity as well as the POST jump (Table 1; Figures 2B, D, 7B). Jump performance returned to normal by the 10th jump after returning to normal gravity (Washout), with jump height, target error, lift impulse, and land impulse not statistically different from PRE (Table 1; Figures 2A–D).

**TABLE 1:** Statistical summary for jump performance metrics and Triceps Surae Preactivity.

	*df*	*t-stat*	*z value*	*p*	*Effect Size (Hedges’ g*_*av*_ *)*
**Jump Height**					
*Early Adaptation*	19	--	0.037	.970	0.065
*Late Adaptation*	17	−0.946	--	.357	0.080
*POST*	19	2.615	--	***.017***	0.489
*Washout*	18	1.946	--	.067	0.119
**|Target Error|**					
*Early Adaptation*	19	--	−3.808	***.000***	1.816
*Late Adaptation*	17	--	−0.806	.420	0.286
*POST*	19	--	−3.323	***.001***	1.101
*Washout*	18	0.214	--	.833	0.047
**Lift Impulse**					
*Early Adaptation*	19	8.610	--	***.000***	2.218
*Late Adaptation*	19	--	3.808	***.000***	1.661
*POST*	19	2.903	--	***.009***	0.565
*Washout*	19	−0.641	--	.529	0.079
**Land Impulse**					
*Early Adaptation*	19	11.287	--	***.000***	2.265
*Late Adaptation*	19	8.294	--	***.000***	1.791
*POST*	19	3.198	--	***.005***	0.535
*Washout*	19	−0.252	--	.803	0.037
**Dominant Side**					
***Triceps Surae Preactivity***					
*Early Adaptation*	19	--	3.509	***.000***	1.310
*Late Adaptation*	19	--	3.136	***.002***	0.844
*POST*	19	4.676	--	***.000***	1.132
*Washout*	19	2.850	--	***.010***	0.547
**Non-Dominant Side**					
***Triceps Surae Preactivity***					
*Early Adaptation*	19	4.571	--	***.000***	1.260
*Late Adaptation*	19	--	3.285	***.001***	1.314
*POST*	19	--	3.173	***.002***	1.122
*Washout*	19	3.179	--	***.005***	0.641

All comparisons made relative to PRE. *p*-values < .05 are ***bolded*.**

### Effect of simulated hypogravity on muscle preactivation

**FIGURE 3:**
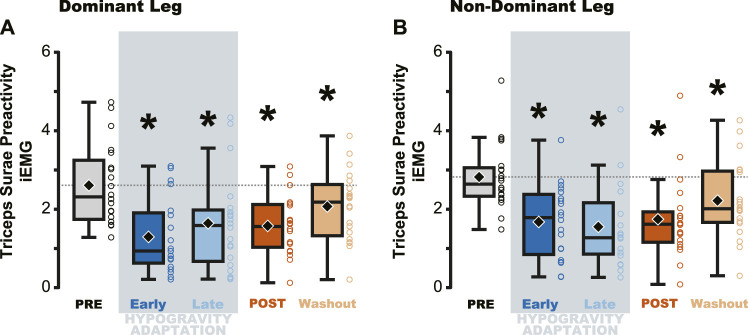
Average muscle preactivity of the entire triceps surae was reduced due to hypogravity exposure. Integrated muscle activation (iEMG) was calculated for each muscle of the triceps surae over the preactivation period and then averaged across all three muscles, resulting in a metric that represents activation of the triceps surae as a whole. Both the dominant **(A)** and non-dominant **(B)** legs showed reductions in Triceps Surae Preactivity during and after hypogravity exposure, with preactivity remaining reduced thru the Washout trial. Boxplot description is provided in the Figure 2 legend.

Triceps Surae Preactivity, a measure of the muscle activation across all three muscles of the triceps surae, was reduced during and following jumping in simulated hypogravity. Compared to PRE, Triceps Surae Preactivity was reduced for both legs during Early and Late Hypogravity Adaptation, as well as upon returning to normal gravity (POST) thru the Washout trial (Figure 3).

**FIGURE 4:**
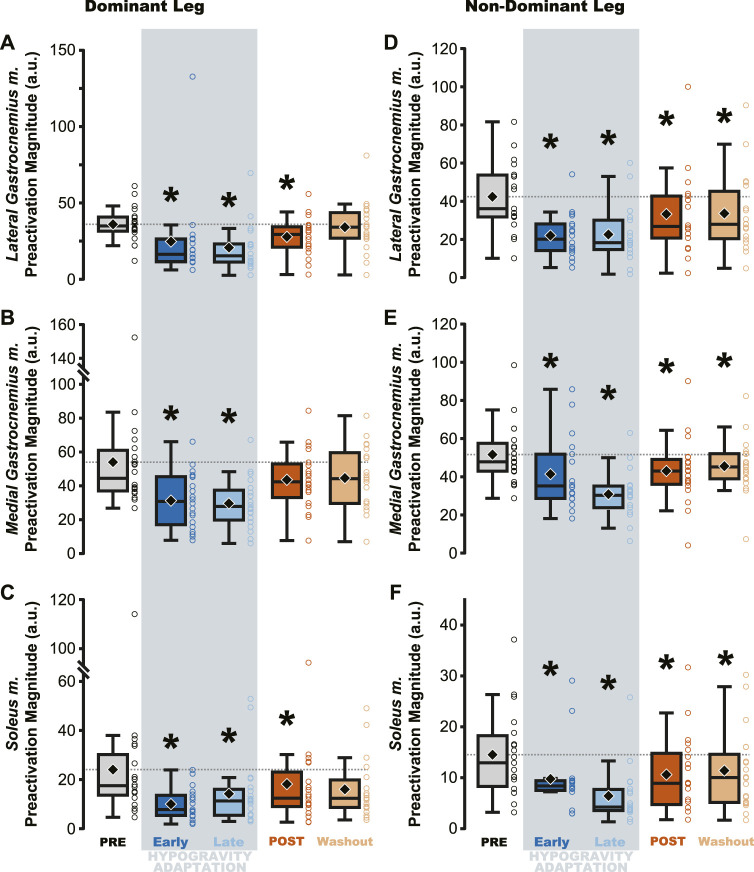
Preactivation magnitude was reduced during and after hypogravity exposure. Muscle preactivation magnitude, or the average muscle activity during preactivation, saw reductions during hypogravity exposure (blue) on the dominant side **(A–C)** and non-dominant side **(D–F)** in all muscles of the triceps surae. This reduction persisted through the first jump upon return to normal gravity (orange), and for some muscles **(C, D, F)** persisted through the Washout jump. Boxplot description is provided in the Figure 2 legend.

Jumping in simulated hypogravity led to reduced preactivation magnitude in the triceps surae muscles (Table 2; Figure 4). As expected, participants preactivated the LG, MG, and SOL prior to landing, with the magnitude of preactivation significantly reduced in all six muscles during exposure to simulated hypogravity, relative to PRE (Table 2; Figure 4). This reduction in preactivation magnitude persisted as an aftereffect in five of the six muscles of the triceps surae (Dominant side: LG and SOL; Non-dominant side: LG, MG, and SOL) after returning to normal gravity (POST; Table 2 and Figure 4; Figure 7). By the Washout jump, three of the six muscles (Non-dominant side: LG, MG, and SOL) still exhibited reduced preactivation magnitude (Table 2; Figure 4).

**TABLE 2:** Statistical summary for muscle preactivation magnitude of individual triceps surae muscles.

*Muscle*	*df*	*t-stat*	*z value*	*p*	*Effect Size (Hedges’ g*_*av*_ *)*
**Dominant Side**					
***Lateral Gastrocnemius m.* (LG)**					
*Early Adaptation*	16	--	2.675	***.007***	0.548
*Late Adaptation*	17	--	3.027	***.002***	1.136
*POST*	19	3.178	--	***.005***	0.681
*Washout*	19	0.560	--	.582	0.135
***Medial Gastrocnemius m.* (MG)**					
*Early Adaptation*	18	--	3.823	***.000***	1.025
*Late Adaptation*	18	--	3.823	***.000***	1.081
*POST*	18	--	1.771	.077	0.430
*Washout*	18	--	1.851	.064	0.352
***Soleus m.* (SOL)**					
*Early Adaptation*	18	--	3.179	***.001***	0.941
*Late Adaptation*	19	--	3.285	***.001***	0.519
*POST*	19	--	2.987	***.003***	0.268
*Washout*	18	--	1.891	.059	0.289
**Non-Dominant Side**					
***Lateral Gastrocnemius m.* (LG)**					
*Early Adaptation*	16	--	3.574	***.000***	1.250
*Late Adaptation*	16	--	3.479	***.001***	1.061
*POST*	17	--	2.635	***.008***	0.457
*Washout*	18	--	2.495	***.013***	0.440
***Medial Gastrocnemius m.* (MG)**					
*Early Adaptation*	17	--	2.417	***.016***	0.582
*Late Adaptation*	17	--	3.506	***.000***	1.375
*POST*	19	--	2.128	***.033***	0.511
*Washout*	18	2.136	--	***.047***	0.437
***Soleus m.* (SOL)**					
*Early Adaptation*	15	--	2.844	***.004***	0.670
*Late Adaptation*	17	--	3.724	***.000***	1.071
*POST*	18	3.468	--	***.003***	0.433
*Washout*	18	2.222	--	***.039***	0.329

All comparisons made relative to PRE. *p*-values < .05 are ***bolded*.**

**FIGURE 5:**
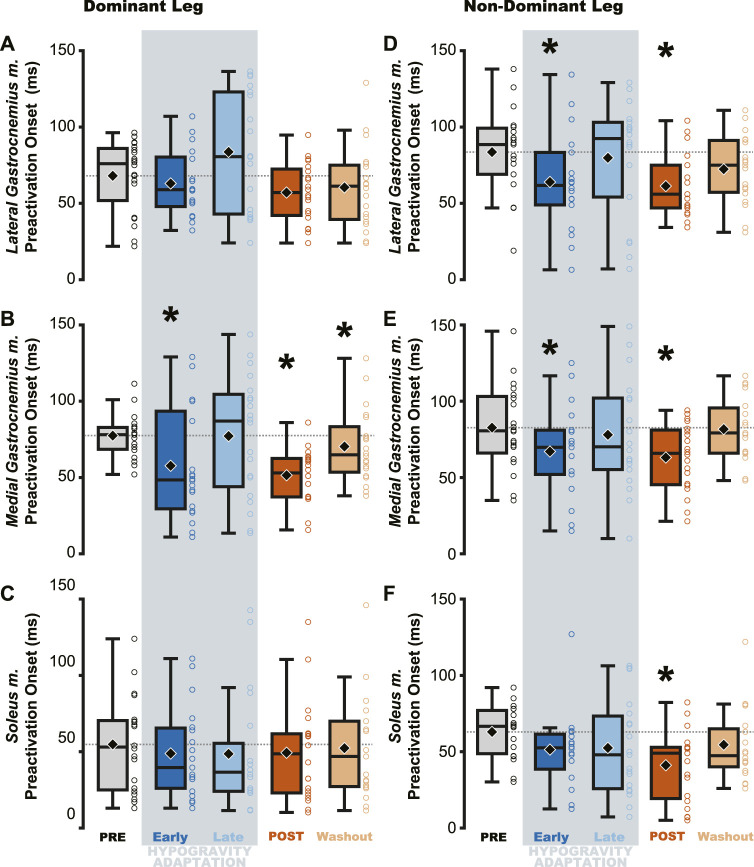
Muscle preactivation onset decreased (i.e., occurred closer to landing) following hypogravity exposure. EMG electrodes were placed bilaterally on the triceps surae and preactivation onset was calculated as the time between muscle activation and landing. The aftereffect of delayed preactivation was observed in all muscles of the non-dominant triceps surae **(D–F)** and in the medial gastrocnemius m. of the dominant-side (B), with no observed effect in the other muscles of the dominant triceps surae **(A, C)**. Boxplot description is provided in the Figure 2 legend.

Changes in preactivation timing were most apparent as an aftereffect of simulated hypogravity exposure, exemplified by a significant delay in preactivation onset upon returning to normal gravity (POST; Table 3 and Figure 5; Figure 8). Prior to exposure to simulated hypogravity, participants preactivated their ankle extensors within a 100 m time window before landing (Table 3; Figure 5). Upon initial exposure to simulated hypogravity (Early Adaptation), the time between preactivation and landing was reduced in three of the six triceps surae muscles (Dominant side: MG; non-dominant side: LG and MG; Table 3 and Figure 5). During the final jump in simulated hypogravity (Late Adaptation), no difference from PRE was observed for preactivation onset in any muscle (Table 3; Figure 5). Upon returning to normal gravity (POST), four of the six triceps surae muscles (Dominant side: MG; non-dominant side: LG, MG, and SOL) exhibited a significant reduction in preactivation onset (Table 3; Figure 5; Figure 8). By the 10th jump in normal gravity (Washout), difference from PRE was only in one muscle (Dominant side: MG; Table 3 and Figure 5).

**TABLE 3:** Statistical summary for muscle preactivation onset of individual triceps surae muscles.

*Muscle*	*df*	*t-stat*	*z value*	*p*	*Effect Size (Hedges’ g*_*av*_ *)*
**Dominant Side**					
***Lateral Gastrocnemius m.* (LG)**					
*Early Adaptation*	16	--	1.060	.289	0.288
*Late Adaptation*	17	−1.117	--	.280	0.380
*POST*	19	--	1.717	.086	0.504
*Washout*	19	--	1.587	.113	0.299
***Medial Gastrocnemius m.* (MG)**					
*Early Adaptation*	18	--	2.274	***.023***	0.721
*Late Adaptation*	18	--	0.161	.872	0.087
*POST*	18	7.139	--	***.000***	1.561
*Washout*	18	2.369	--	***.029***	0.573
***Soleus m.* (SOL)**					
*Early Adaptation*	18	0.502	--	.622	0.138
*Late Adaptation*	19	--	0.989	.322	0.177
*POST*	19	--	1.045	.296	0.169
*Washout*	18	0.263	--	.796	0.074
**Non-Dominant Side**					
***Lateral Gastrocnemius m.* (LG)**					
*Early Adaptation*	16	2.183	--	***.044***	0.524
*Late Adaptation*	16	--	0.260	.795	0.077
*POST*	17	3.016	--	***.008***	0.979
*Washout*	18	1.961	--	.066	0.429
***Medial Gastrocnemius m.* (MG)**					
*Early Adaptation*	17	2.316	--	***.033***	0.509
*Late Adaptation*	17	0.260	--	.798	0.043
*POST*	19	4.004	--	***.001***	0.772
*Washout*	18	0.729	--	.475	0.146
***Soleus* (SOL)**					
*Early Adaptation*	15	--	1.215	.224	0.506
*Late Adaptation*	17	--	1.590	.112	0.460
*POST*	18	3.975	--	***.001***	1.013
*Washout*	18	--	1.590	.112	0.388

All comparisons made relative to PRE. *p*-values < .05 are *bolded*.

### Effect of simulated hypogravity on muscle activity and estimated proprioceptive feedback during lift, aerial, and landing phases

**FIGURE 6:**
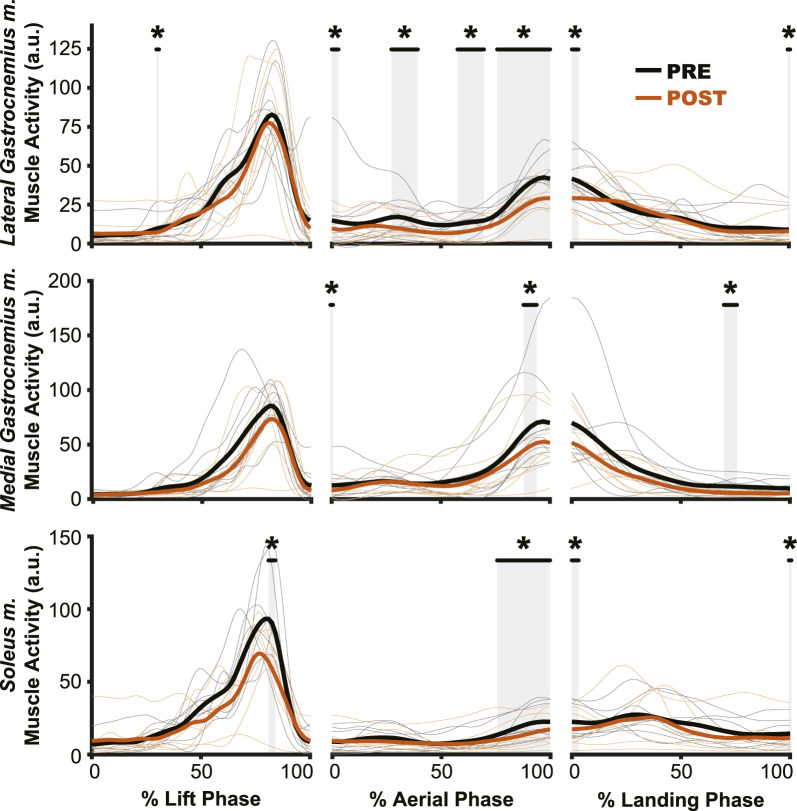
Muscle activation changes after exposure to hypogravity were largely absent outside of the preactivation phase. The primary differences in muscle activation waveforms (*n* = 10) between PRE and POST occurred during the aerial phase (middle column), especially close to the time of landing. Intermittent, transient differences were observed during the lift (left column) and land (right column) phases. PRE = black, POST = orange, significant difference between PRE and POST = grey highlight. Individual participant data shown in thinner lines.

**FIGURE 7:**
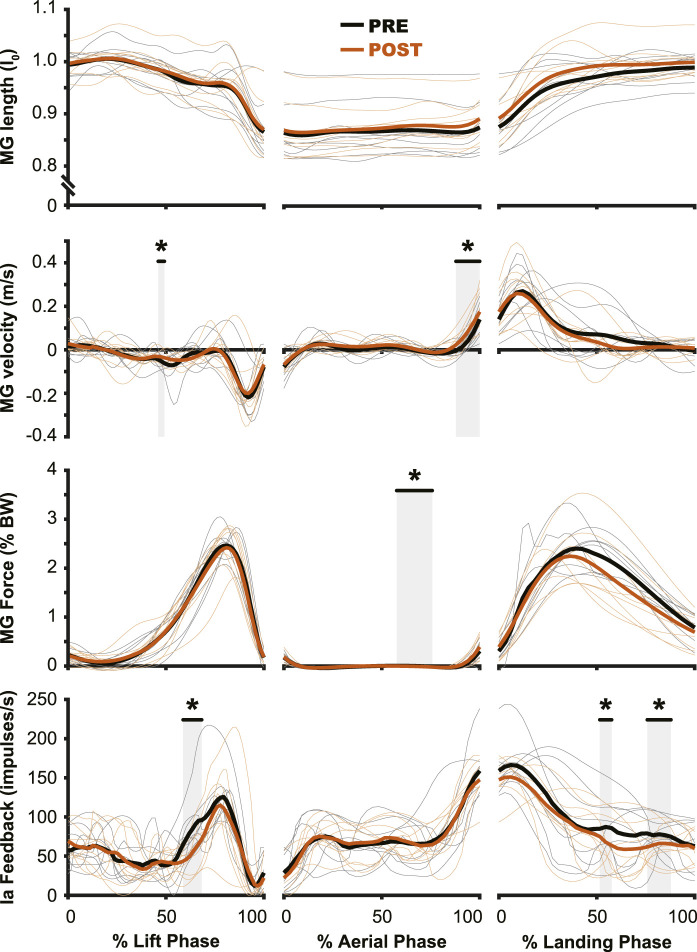
Muscle dynamics of the *medial gastrocnemius m.* (MG) changed very little from PRE to POST. While no changes were observed in muscle length (top row), muscle velocity (second row) saw similar changes to those of muscle activation (Figure 5), where the main change was observed during the preactivation phase. The estimated muscle spindle feedback was relatively unchanged for the aerial (middle column) and landing (right column) phases. In the lift phase (left column) estimated muscle spindle feedback was reduced in PRE during the redirection of the center of mass. PRE = black, POST = orange, significant difference between PRE and POST = grey highlight. Individual participant data shown in thinner lines.

The aftereffects of exposure to simulated hypogravity were reflected in the muscle activation and muscle dynamics primarily during the aerial phase, with minimal aftereffects observed when the participant was in contact with the ground. Muscle activity exhibited peaks during the end of the lift phase (i.e., generating force for the jump) and the end of the aerial phase (i.e., preactivation). After experiencing simulated hypogravity, muscle activation during the late aerial phase was reduced in the MG, LG, and SOL but muscle activation during the lift and land phases was mostly unchanged (Figure 6). The whole muscle length, velocity, and force of the MG through most of the jump were unchanged in POST, though muscle velocity just prior to ground contact was higher in POST than in PRE. Muscle force also showed a significant difference between PRE and POST over a small portion of the aerial phase, but both force magnitudes at this time were observed to be negligibly small. The estimated feedback from muscle spindles in the MG followed a similar pattern to that of muscle activation (Figure 6), with increases prior to take-off and around the time of landing (Figure 7). However, no difference in estimated spindle feedback was observed between PRE and POST during aerial and landing phases. A brief difference was observed during the lift phase, corresponding to the time when muscle activity is increasing.

## Discussion

Sensorimotor adaptation to hypogravity represents a unique challenge to the nervous system, and the aim of the current study was to characterize how motor control is affected during simulated hypogravity adaptation and after returning to normal gravity. By having participants perform targeted countermovement jumps before, during, and after physical simulation of hypogravity, we were able to investigate the interplay between predictive and reactive neuromuscular control strategies. For example, when the person is in contact with the ground (lift and land phases) rich proprioceptive feedback can augment the control strategy whereas when the person is airborne, less mechanical sensation is available, leading to an increased reliance on predictive control strategies. We found that the aspects of jumping behavior that most heavily rely on predictive neuromuscular control were most impacted by hypogravity adaptation. Specifically, jump height, target error, and muscle preactivation, all of which occur during the aerial phase, were affected during and following simulated hypogravity exposure, but muscle activation outside of the aerial phase was relatively unchanged. These findings support the hypothesis of a prediction of gravity that underpins predictive locomotor movements and is adapted in response to hypogravity exposure.

### Adaptation to simulated hypogravity is exemplified by aftereffects in predictive control

**FIGURE 8:**
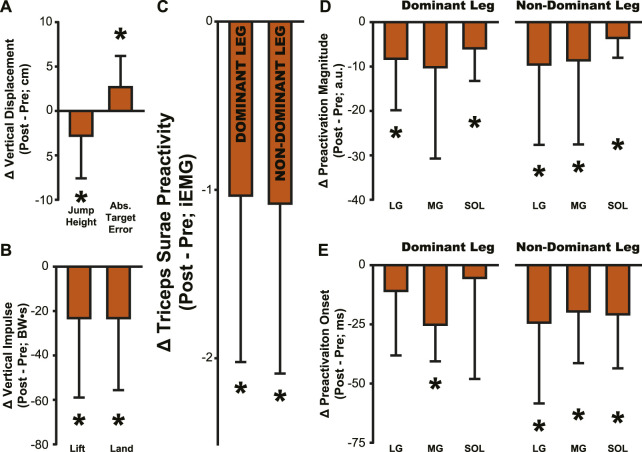
Summary of aftereffects in jump performance and muscle activity. Aftereffects were observed in **(A)** jump height and target error, **(B)** lift and land impulse, **(C)** Triceps Surae Preactivity, **(D)** muscle preactivation magnitude, and **(E)** muscle preactivation onset. All bars represent the PRE value subtracted from the POST value, then averaged across participants. Error bars indicate one standard deviation. Asterisks indicate a significant difference from zero, *p* < .05, a comparison that is identical to the comparisons reported in Tables 1–4 for POST.

The adaptation to simulated hypogravity is perhaps most obvious when looking at the participants’ jumping performance. The participants’ ability to accurately target their vertical jumps (i.e., Abs. Target Error) was impacted following a change to simulated gravity and upon returning to normal gravity (Table 1; Figure 2C; Figure 8A). Similar to what has been observed in other adaptation paradigms that involve walking (Reisman et al., 2005; Selgrade et al., 2017) and reaching (Harris, 1963; Shadmehr and Mussa-Ivaldi, 1994; Tsay et al., 2021), the aftereffects in target error clearly indicate adaptation of jump performance to the lower gravity level. Target error is determined by the force imparted on the ground during the jump. While a participant is airborne, there is little they can do to correct their trajectory. Accordingly, the lift impulse remained reduced following a return to normal gravity (Table 1; Figure 2B; Figure 8B), indicating that the participants expected a lower force to be sufficient to hit the target. The countermovement of the jump was the first dynamic movement that participants engaged in after exiting simulated hypogravity. Therefore, the lift impulse and subsequent target error clearly reveal the newly updated prediction of hypogravity, as they occurred before the participant had any substantial sensorimotor experience upon return to the normal Earth’s gravity level.

The ability to predict the effects of gravity likely serves to prevent injury to the lower limb when jumping as evidenced by the preparatory preactivation of leg muscles prior to landing from the jump. Muscles are especially prone to injury when they experience unexpected, rapid lengthening (Lieber and Friden, 1993; Proske and Morgan, 2001), which many of the leg’s muscles can experience upon landing. One strategy to mitigate injury could be to rely on short-latency reflex pathways to reactively shorten the muscles in response to stretch upon landing (Liddell and Sherrington, 1925; Nichols et al., 2014). However, the so-called stretch reflex incurs a finite delay before it can generate muscle force in response to stretch (Jones and Watt, 1971). When landing from a jump, this delay would allow a brief period of rapid, injurious muscle lengthening. Alternatively, muscle preactivation prior to landing functions to pre-shorten the muscles to anticipate and mitigate the lengthening that would otherwise occur after ground contact (Azizi and Abbott, 2013). This function makes muscle preactivation an important contributor to safely accommodate landing forces.

Despite the importance of proper preactivation, the results of the current study indicate that triceps surae preactivation is reduced following a return to normal gravity from simulated hypogravity. Muscle preactivation can be adapted by changing when the muscles are activated (i.e., Preactivation Onset), the level to which they are activated (i.e., Preactivation Magnitude), or both. We found that overall activation of the plantarflexors (i.e., Triceps Surae Preactivity; Figure 3) was reduced for both legs. Triceps Surae Preactivity was calculated as average of the integrated EMG signal over the preactivation phase across the three muscles of the triceps surae, and so represents a comprehensive metric of preactivation for each leg. However, integrated EMG is susceptible to changes in both Preactivation Onset and Preactivation Magnitude, leading us to also investigate how these changed separately, starting with Preactivation Magnitude. Lower preactivation magnitude of the leg extensors has been shown to coincide with lower expected landing forces (Arampatzis et al., 2003) and reduced kinetic energy of the center of mass at touchdown (Gambelli et al., 2016). Similar scaling of preactivation magnitude can clearly be seen in the current study, where the trials during exposure to simulated hypogravity had greatly reduced landing impulse along with greatly reduced preactivation magnitude (Figure 8). In accordance with our hypothesis, this reduction in muscle preactivation magnitude in hypogravity persisted upon returning to normal gravity, indicating that participants had adapted to expect lower forces upon landing. As stated previously, muscle preactivation is important for injury prevention, so the observed reduction in preactivation magnitude may indicate an increased likelihood of injurious muscle stretch upon landing. However, ultrasound recordings of the MG muscle from a subset of our participants show that muscle length and velocity were unchanged following simulated hypogravity exposure (Figure 7). Even so, precautions should be taken when people return from exposure to hypogravity environments to mitigate the potential increased risk of injury.

We interpret the reduction in preactivation magnitude after returning from simulated hypogravity (POST) to reflect an erroneous expectation of hypogravity even though the participant was jumping in normal gravity. An alternative explanation might point to the reduced jump height and landing impulse, and so interpret the reduced preactivation as actually representing a correct prediction of the force of landing. However, if this were the case, we might expect the change in landing impulse from before simulated hypogravity exposure (PRE) and after returning from simulated hypogravity (POST) to correspond with the change in muscle preactivation magnitude. No correlation was observed between the change in landing impulse and any muscle’s change in preactivation magnitude. Additionally, lift and landing force impulse returned to baseline levels by the final jump (Figure 2, Washout), while three ankle extensor muscles continued to exhibit reduced preactivation magnitude (Figure 4, Washout), indicating that lift and landing mechanics are not the sole driver of muscle preactivation. As such, it appears that the reduction in preactivation magnitude is in line with previous research on drop jumps with artificially decreased vertical acceleration (Avela et al., 1996; Waldvogel et al., 2021), despite our participants having returned to normal gravity. This aftereffect is similar to that observed by Johansson and Westling, where they manipulated the weight of an object that was repeatedly lifted by the participants and showed that the expected weight (informed by the previous lift) was apparent in the force and EMG data (Johansson and Westling, 1988). Similarly, we interpret the observed reductions in ground impulses and preactivation magnitude as an expectation of lower gravity.

While muscle preactivation magnitude during hypogravity exposure saw substantial, consistent decreases, preactivation timing was much more variable across subjects. Preactivation timing was consistent prior to hypogravity exposure, but we observed a large increase in preactivation timing variability throughout the exposure to simulated hypogravity. However, this non-directional increase in variability during hypogravity exposure was followed by a unidirectional aftereffect in preactivation timing (POST, Figure 4; 8). Prior research on the effect of altered gravity on preactivation timing is sparse, but reports a reduction in preactivation onset with reduced vertical acceleration during drop landings (Gollhofer and Kyröläinen, 1991; Avela et al., 1996). As such, we expected a similar reduction during simulated hypogravity exposure, but this was not observed. The apparent incongruence between previous results and the current study likely lies in methodological differences, including the current study’s use of countermovement jumps as opposed to drop jumps, using constant-force springs to offload participants rather than counterweights, and differences in criteria for determining muscle activation onset. We were as careful and systematic as possible with our calculation of preactivation onset, but muscle activation levels were quite low during hypogravity exposure which made determination difficult, prompting us to omit trials that did not meet specific criteria (see Method - *Data Analysis: Electromyography*). Thus, we are confident that the reported data reflect the actual preactivation times of our participants. One reason for the relatively low preactivation magnitudes could be that countermovement jumps show lower preactivation magnitudes than drop jumps (McBride et al., 2008), and this is likely further exacerbated by the exposure to a lower force requirement in simulated hypogravity. There are many important differences between drop jumps and countermovement jumps, and the current study adds to this list the observation that preactivation onset is not reduced when landing from countermovement jumps in simulated hypogravity. Despite this, we still observed a significant delay in preactivation timing as an aftereffect upon returning to normal gravity after exposure to hypogravity.

More specifically, upon returning to normal gravity after hypogravity adaptation, muscle preactivation time was reduced by 28.3%–33.0%. Successful muscle preactivation timing relies on an accurate prediction of the acceleration due to gravity so that the timing and magnitude of the activation are appropriate for a given landing. Normally, preactivation is tightly controlled to occur at a specific time prior to landing, even in the face of different fall times and heights (Greenwood and Hopkins, 1976a; Greenwood and Hopkins, 1976b; Santello and Mcdonagh, 1998; Santello et al., 2001; Niu et al., 2011). For example, the SOL muscle is consistently activated ∼100–120 m prior to landing from a drop jump, even when the drop height ranges from 0.2–1.2 m (Santello and Mcdonagh, 1998). Maintaining such a precise timing regardless of initial drop height (and thereby aerial time) speaks to a sophisticated prediction of the time of landing. The delayed preactivation observed in the current study reflects the expectation of hypogravity, even though the participants were well-aware they had returned to jumping in normal gravity. However, it does not appear that the participants were fully expecting the gravity to which they had been exposed. If we consider the average jump height of 27.3 cm (Figure 2A, POST), the time to fall from that height will be approximately 236 ms under 1 g and 331 ms under 0.508 g (the average hypogravity simulated in this study), with the difference being 95 ms. If preactivation timing is as tightly synced to the landing time as has been previously suggested and the expectation of the imposed hypogravity level was perfectly maintained, we would then expect a delay in preactivation of 95 ms relative to the PRE preactivation time. Instead, we see an average delay closer to 26 ms in the dominant-side MG, for example,. We interpret this discrepancy as arising from the interplay between feedback and feedforward systems. Rich sensory feedback is being provided during the initiation and aerial phase of the jump, providing cues that the participant has returned to normal gravity. These cues are not sufficient to fully override the feedforward expectation of lower gravity, but are still strong enough to reduce the timing delay from the predicted 95 ms to the observed 26 ms, or by about 72%. While it is beyond the scope of this study to determine the percentage contributions of feedback and feedforward systems to a particular behavior, this brief analysis speaks to both the strength of the feedforward expectation and fast utilization of the incoming feedback.

The primary contribution of the current study is the strong evidence that expectations of gravity are updated in response to simulated hypogravity exposure. This has been speculated in the past, but the confirmation of a newly adapted, feed-forward prediction through the investigation of neuromotor aftereffects has not been fully explored. The increased performance error, reduced ground impulse, and the reduced and delayed muscle preactivation exemplify the prediction of hypogravity despite returning to normal gravity. As such, our results agree with previous suggestions of an internal model of gravity that is updated based on recent experience (Zago et al., 2005; Torok et al., 2019).

### Reactive control and sensory mechanisms contributing to simulated hypogravity adaptation

The muscle spindle is an important proprioceptor, as it provides information on muscle dynamics to the central nervous system as well as more directly affecting behavior through spinal networks (Sherrington, 1894; Ellaway et al., 2015; Blum et al., 2017). Sensory information from the muscle spindle, which is primarily sensitive to changes in muscle length and velocity, is carried through the group Ia and group II afferent fibers to the spinal cord, where it can directly increase the activation of the muscle in which they reside, affect other muscles, and be transmitted supraspinally. The muscle spindle’s sensitivity to changes in muscle contraction make it an incredibly important proprioceptor and a likely progenitor for the adaptation that is seen during hypogravity exposure. However, previous work leveraging the Hoffman reflex (an electrical stimulation analogue of the spindle-induced stretch reflex) to study spinal circuits during hypogravity locomotion found that the strength of the Hoffman reflex was not impacted by reductions in gravity level (Ferris et al., 2001). Similarly, we observed little change in estimated muscle spindle feedback over most of the countermovement jump and landing, with the exception of the propulsive portion of the jump (Figure 7).

When jumping at normal gravity after exposure to hypogravity, estimated muscle spindle feedback was reduced for a small duration of the lift phase of the jump, coinciding with the center of mass reaching its lowest point of the countermovement. This was also the time of the lift phase where EMG activity and muscle force began to rapidly increase. Despite this change in muscle spindle feedback, no change was observed in the magnitude of muscle activation during the lift (Figure 7). This lack of change may be because the muscle activity is already high during a jump, so the contribution of the stretch reflex is harder to detect at the muscle activation level (Van Hooren and Zolotarjova, 2017). However, as mentioned previously, the muscle spindle also sends afferent signals supraspinally. So, while the change in muscle spindle feedback does not manifest in an immediate change in muscle activity, it may contribute to an altered perception of the movement. In fact, open-ended questioning of the participants upon returning to normal gravity revealed a prolonged sense of “heavy legs,” where participants indicated a “higher effort” was required to accomplish the jump. All 20 participants reported such a feeling for the first jump after returning from simulated hypogravity, with the feeling still present during the fifth jump for 18 participants, and during the 10th jump (i.e., the final jump of the experiment) for 11 participants. While it is speculative to attribute the origin of this self-reported altered perception, we do not think it originates from the muscle spindle, which was ostensibly unchanged for most of the movement. Instead, we expect that the nervous system’s expectation of the proprioceptive feedback may have changed and, when faced with “normal” sensory input, interprets this mismatch as “heaviness”.

Proprioceptors in the leg muscles are incredibly important sensory organs for motor adaptation but they are certainly not the only sensory system impacted by our protocol. Vision, vestibular function, and proprioception from other sources are all potentially important sources of feedback for informing neuromuscular adaptation to simulated hypogravity. In the current study, each of these systems was most likely signaling the “correct” gravity level (i.e., hypogravity in the simulated hypogravity condition and normal gravity otherwise). Optic flow, which is important for locomotor control (Warren et al., 2001) and adaptation (Finley et al., 2014), was certainly slower in the simulated hypogravity condition. Similarly, the viscous fluid of the otoliths would have experienced reduced acceleration in the simulated hypogravity condition. Proprioceptors in the legs experienced the lower force of the simulated hypogravity condition, and their estimated activity was unchanged for most of the duration of the movement between PRE and POST (Figure 7). During simulated hypogravity exposure, movement of the arms was minimized by having participants cross their arms over their chest throughout the protocol. Without movement, the proprioceptive organs of the arm muscles would not have been providing much sensory input. As such, it appears that each of these sensory systems was experiencing and encoding similar environments to each other. In other words, there was little-to-no opportunity for sensory conflict, where different sensory systems encode incongruously different phenomena. Instead, it appears that the conflict arises from comparing the anticipated and actual sensory input. As stated above, the observed aftereffects in preactivation dynamics did not fully reflect an expectation of the enforced gravity level. As such, the proprioceptive, vestibular, visual, and other sensory input are able to rapidly mitigate the size of the aftereffect. While the current study is unable to parse which of these sensory systems is responsible for the adaptation to or de-adaptation from hypogravity, it remains an outstanding area for future work.

### Neuromechanical adaptation in the nervous system

When describing how gravity is represented by the nervous system, the concept of the internal model is often used (Merfeld et al., 1999; Zago et al., 2005; Lacquaniti et al., 2014; Rousseau et al., 2021). In general, internal models are neural representations of physical or abstract phenomena, are thought to exist in the cerebral cortex (Rousseau et al., 2021) and cerebellum (Wolpert et al., 1998; Imamizu et al., 2000), and are important for predicting motion and generating motor commands (Wolpert et al., 1998). For example, internal models are thought to be employed by the central nervous system to perceive one’s body and movement (Wolpert et al., 1995) and to facilitate interaction with objects (Flanagan and Wing, 1997; Flanagan et al., 2001). Internal models related to gravity may be thought of as a form of physics engine used to make accurate predictions of how objects will act in a gravitational environment (Battaglia et al., 2013; Ullman et al., 2017). Neuroimaging and brain stimulation studies have identified the insular cortex and temporo-parietal junction as likely candidates for housing the internal model of gravity (Isnard et al., 2004; Indovina et al., 2005; Bosco et al., 2008; Maffei et al., 2015), with additional gravity-dependent activity in visual and motor areas (Cignetti et al., 2017) as well as the cerebellum (Laurens et al., 2013; MacNeilage and Glasauer, 2018; Mackrous et al., 2019). Investigations on movement and perception of gravity have revealed a reliance on an assumption of normal Earth gravity (McIntyre et al., 2001), but this assumption can be altered with time and training (Crevecoeur et al., 2009; Torok et al., 2019), suggesting that the internal model of gravity is adaptable. In fact, a recent model of the insular cortex suggests how the internal model of gravity may achieve adaptation, with sensory feedback being received and modelled by the posterior insula, which then informs the feedforward predictions of the anterior insula (Rousseau et al., 2021).

The results of the current study indicate an updated internal model of gravity, as participants experienced aftereffects in their predictive neuromuscular control of the legs after only a brief exposure to simulated hypogravity. In the context of motor adaptation to a new task, existing internal models may be updated to accommodate a new task, or an entirely new model may be generated (Imamizu et al., 2000). Previous examples of internal models being generated and/or adapted include using a computer mouse to track a target with the movement of the cursor rotated 120° from the input (Imamizu et al., 2000) as well as using prism goggles to shift the visual field during a targeted arm movement (Petitet et al., 2018). In these examples, it was suggested that a new internal model may have been created due to the novel relationship between the movement and the sensory feedback. In the current study, the general relationships between the movement, the direction of force application, and the sensory feedback were relatively consistent whether the participant was in normal or hypogravity. As such, we might infer that the existing internal model of gravity was simply updated, as opposed to a new model being created for hypogravity. This interpretation meshes well with our current understanding of hypogravity and microgravity adaptation, which have both been previously studied to shed light on how the internal model of gravity responds to different environments. When adapting to move in microgravity (g ∼ 0.0 m/s^2^), normal modes of locomotion become impossible; instead, we see astronauts floating in a prone position, pushing themselves from place to place. This completely new form of locomotion must build a new understanding of the relationship between the sensory and motor systems to guide successful movement. The novelty of microgravity locomotion makes it extremely likely that a new internal model is created and used for movement. This may also help explain why the expectation of gravity in certain tasks takes a long time to change in microgravity. For example, multiple weeks of constant microgravity exposure is insufficient to fully override the expectation of normal gravity in a catching task (McIntyre et al., 2001). In other words, tasks that typically rely on an internal model of gravity (i.e., catching) are enacted with the expectation of Earth gravity, despite continuous exposure to microgravity, indicating that the internal model is maintained despite a new internal model being developed for other microgravity tasks, like locomotion. This maintenance of an Earth gravity ‘prior’ over weeks speaks to the strength of the internal model of normal gravity; without task-specific sensory information being received, the internal model does not appear to decay or change. In contrast, experience in hypogravity (0 < g < 9.81 m/s^2^) allows for modes of locomotion, as well as the associated relationships between sensory and motor systems, that are more similar to those in normal gravity. The internal model of gravity appears to quickly adapt to hypogravity, whether it be changes in muscle dynamics during jumping (Monti et al., 2021) or predicting the movement of a virtual ball in different gravity levels (Torok et al., 2019). The relatively rapid adaptation to hypogravity (when compared to microgravity) indicates that even though the existing internal model of gravity is strong, it can be updated when faced with a new context-specific sensory input. In agreement with existing research, the current study shows a quick adaptation to hypogravity occurring within 50 targeted jumps, as exemplified by the participants’ reduction in target error. The quick adaptation to accurate jumping performance in simulated hypogravity, along with the aftereffects upon returning to normal gravity (Figure 8), provide strong evidence that the internal model of gravity was updated in response to exposure to simulated hypogravity.

## Conclusion

Motor adaptation relies on predictions related to the environment and sensory feedback. By imposing a physical simulation of hypogravity, we aimed to determine if the prediction of gravity is updated in response to motor experience in altered gravity. We hypothesized the internal model of gravity would be updated such that performance would be affected both during and following exposure to simulated hypogravity. Consistent with our hypothesis, the portions of the countermovement jump that most rely on a prediction of gravity were affected, such as jump height and muscle preactivation prior to landing. These results point to an updated internal model of gravity that was able to change over a relatively short exposure to simulated hypogravity. Furthermore, reactive mechanisms appear insufficient to account for the observed aftereffects, reflected by our measurement of muscle dynamics and calculated estimation of muscle spindle feedback that were largely unchanged before and after adaptation to simulated hypogravity. Thus, our results support the existence of an internal model of gravity that rapidly updates in the face of new context specific sensory cues. The extent to which this internal model of gravity is malleable remains to be seen, both in regards to its ability to accommodate different ranges of gravity levels or its ability to generalize the adaptation to other gravity-dependent tasks.

## Data Availability

The original contributions presented in the study are included in the article/Supplementary material, further inquiries can be directed to the corresponding author.
